# The Anti-Senescence Activity of Cytokinin Arabinosides in Wheat and Arabidopsis Is Negatively Correlated with Ethylene Production

**DOI:** 10.3390/ijms21218109

**Published:** 2020-10-30

**Authors:** Zuzana Kučerová, Marek Rác, Jaromír Mikulík, Ondřej Plíhal, Pavel Pospíšil, Magdaléna Bryksová, Michaela Sedlářová, Karel Doležal, Martina Špundová

**Affiliations:** 1 Department of Biophysics, Centre of the Region Haná for Biotechnological and Agricultural Research, Faculty of Science, Palacký University, Šlechtitelů 27, CZ-78371 Olomouc, Czech Republic; zuzana.kucerova@upol.cz (Z.K.); marek.rac@upol.cz (M.R.); pavel.pospisil@upol.cz (P.P.); 2 Laboratory of Growth Regulators, Faculty of Science, Palacký University and Institute of Experimental Botany AS CR, Šlechtitelů 27, CZ-78371 Olomouc, Czech Republic; jaromir.mikulik@upol.cz (J.M.); ondrej.plihal@upol.cz (O.P.); karel.dolezal@upol.cz (K.D.); 3 Department of Chemical Biology and Genetics, Centre of the Region Haná for Biotechnological and Agricultural Research, Faculty of Science, Palacký University, Šlechtitelů 27, CZ-78371 Olomouc, Czech Republic; magdalena.bryksova@upol.cz; 4 Department of Botany, Faculty of Science, Palacký University, Šlechtitelů 27, CZ-78371 Olomouc, Czech Republic; michaela.sedlarova@upol.cz

**Keywords:** cytokinin derivative, ethylene, senescence, chlorophyll fluorescence, wheat, Arabidopsis, phytohormone, oxidative stress, photosystem II

## Abstract

Leaf senescence, accompanied by chlorophyll breakdown, chloroplast degradation and inhibition of photosynthesis, can be suppressed by an exogenous application of cytokinins. Two aromatic cytokinin arabinosides (6-benzylamino-9-β-d-arabinofuranosylpurines; BAPAs), 3-hydroxy- (3OHBAPA) and 3-methoxy- (3MeOBAPA) derivatives, have recently been found to possess high anti-senescence activity. Interestingly, their effect on the maintenance of chlorophyll content and maximal quantum yield of photosystem II (PSII) in detached dark-adapted leaves differed quantitatively in wheat (*Triticum aestivum* L. cv. Aranka) and Arabidopsis (*Arabidopsis* *thaliana* L. (Col-0)). In this work, we have found that the anti-senescence effects of 3OHBAPA and 3MeOBAPA in wheat and Arabidopsis also differ in other parameters, including the maintenance of carotenoid content and chloroplasts, rate of reduction of primary electron acceptor of PSII (Q_A_) as well as electron transport behind Q_A_, and partitioning of absorbed light energy in light-adapted leaves. In wheat, 3OHBAPA had a higher protective effect than 3MeOBAPA, whereas in Arabidopsis, 3MeOBAPA was the more efficient derivative. We have found that the different anti-senescent activity of 3OHBAPA and 3MeOBAPA was coupled to different ethylene production in the treated leaves: the lower the ethylene production, the higher the anti-senescence activity. 3OHBAPA and 3MeOBAPA also efficiently protected the senescing leaves of wheat and Arabidopsis against oxidative damage induced by both H_2_O_2_ and high-light treatment, which could also be connected with the low level of ethylene production.

## 1. Introduction

Phytohormones cytokinins (CKs) have a crucial role in almost all stages of plant growth and development, including senescence. Senescence is a highly organized and controlled process that is characterized by dramatic structural and functional changes, such as degradation of photosynthetic pigments and inhibition of photosynthesis (e.g., [1,2,3]), and by extensive changes in gene expression [4]. An exogenous application of CKs typically delays senescence-induced decreases in chlorophyll (Chl) and carotenoid content, inhibition of photosynthesis, degradation of proteins, pigment–protein complexes and whole chloroplasts, as well as membrane deterioration (e.g., [3,5,6,7,8,9]). The anti-senescence activity of CKs is related to the downregulation of senescence-associated genes (*SAG*s) [10] and upregulation of genes encoding components of photosynthetic light-harvesting complexes [11].

From an agricultural point of view, leaf senescence is one of the major traits that negatively affects crop production, and therefore the delay of this process represents an ideal target for improving crop yield and quality [12]. One of the natural CKs that is used both in agriculture [13] and biotechnology is the aromatic CK 6-benzylaminopurine (BAP), mainly because of its activity, affordability, and stability in plants [14,15]. BAP has significant anti-senescence activity and its exogenous application effectively delays senescence-induced changes (e.g., [3,8,9]). However, under certain conditions, BAP can have negative effects on primary root development, lateral root branching, or shoot formation in vitro (e.g., [16,17,18,19]). When applied at higher concentrations or in combination with light, BAP can even accelerate senescence [11,20,21]. These findings led to the search for aromatic CK derivatives that would not exhibit these negative effects on plant growth and development [22]. Several previously prepared compounds have been shown to be more efficient in delaying senescence than unmodified BAP, including those with hydroxy- or methoxy-groups introduced to the benzyl ring (e.g., [5,23,24]).

**Figure 1:**
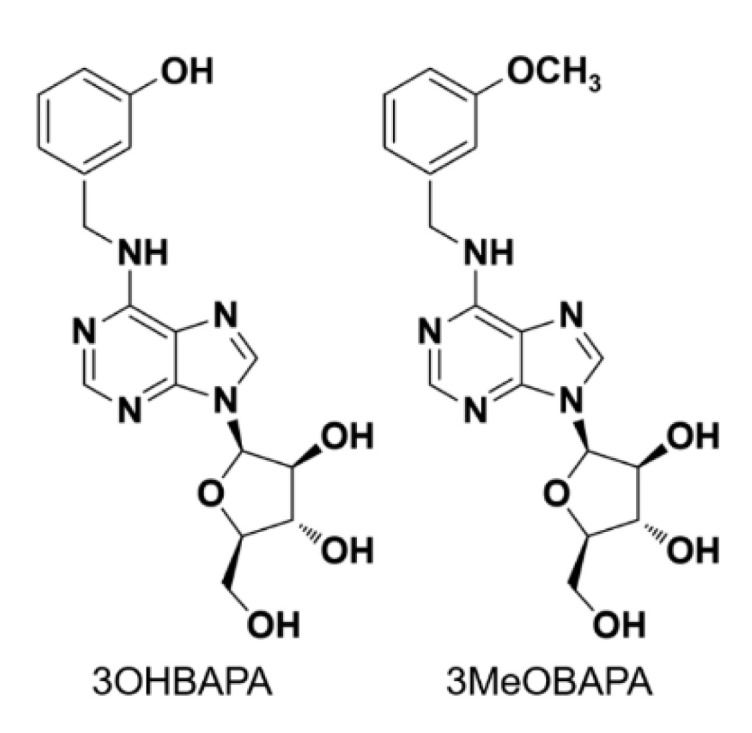
Structure of 6-(3-hydroxybenzylamino)-9-β-d-arabinofuranosylpurine (3OHBAPA, left) and 6-(3-methoxybenzylamino)-9-β-d-arabinofuranosylpurine (3MeOBAPA, right).

In our previous work, novel 6-benzylamino-9-β-d-arabinofuranosylpurines (BAPAs) were synthesized [25] and their unique mode of action has been described [26]. BAPAs have high anti-senescence activity, but they have generally low interactions with the CK pathway and thus also very low activity in other CK-regulated processes and no negative effects on root/shoot development [26]. We have observed that the application of BAPAs induced a massive transcriptional reprogramming from photosynthesis toward defense responses, including the elicitation of several MAPK modules, suggesting an induction of a pathogen-associated molecular patterns (PAMP)-like response [26]. This response was accompanied by delaying senescence. A comparison of the anti-senescence activity of two selected BAPAs, 3-hydroxy- (3OHBAPA) and 3-methoxy- (3MeOBAPA) derivatives (see Figure 1), indicated that their protective effect during senescence (maintenance of Chl content and maximal quantum yield of photosystem II (PSII)) differs quantitatively in wheat and Arabidopsis: 3OHBAPA had higher protective effect in wheat, while in Arabidopsis 3MeOBAPA was more efficient. 

Here, we have investigated whether the different effects of 3OHBAPA and 3MeOBAPA on the senescence of detached wheat (*Triticum aestivum* L. cv. Aranka) and *Arabidopsis thaliana* L. (Col-0) leaves will also manifest in the maintenance of other parameters related to photosynthetic apparatus and its function. We have evaluated the changes in carotenoid content and chloroplasts, rate of reduction of primary electron acceptor of PSII (Q_A_) and electron transport behind Q_A_, and changes in the partitioning of absorbed light energy in light-adapted leaves. We have also explored the possibility that the different anti-senescent activity of 3OHBAPA and 3MeOBAPA in both plant models is related to a different level of ethylene production in the treated leaves, as ethylene is known to co-determine the progression of senescence in plant samples treated by anti-senescence compounds [27]. Finally, we have evaluated whether 3OHBAPA and 3MeOBAPA can protect senescing leaves of wheat and Arabidopsis against oxidative damage induced by both H_2_O_2_ and high-light treatment.

## 2. Results and Discussion

### 2.1. Senescence-Induced Changes in Detached Leaves of Wheat and Arabidopsis

**Figure 2:**
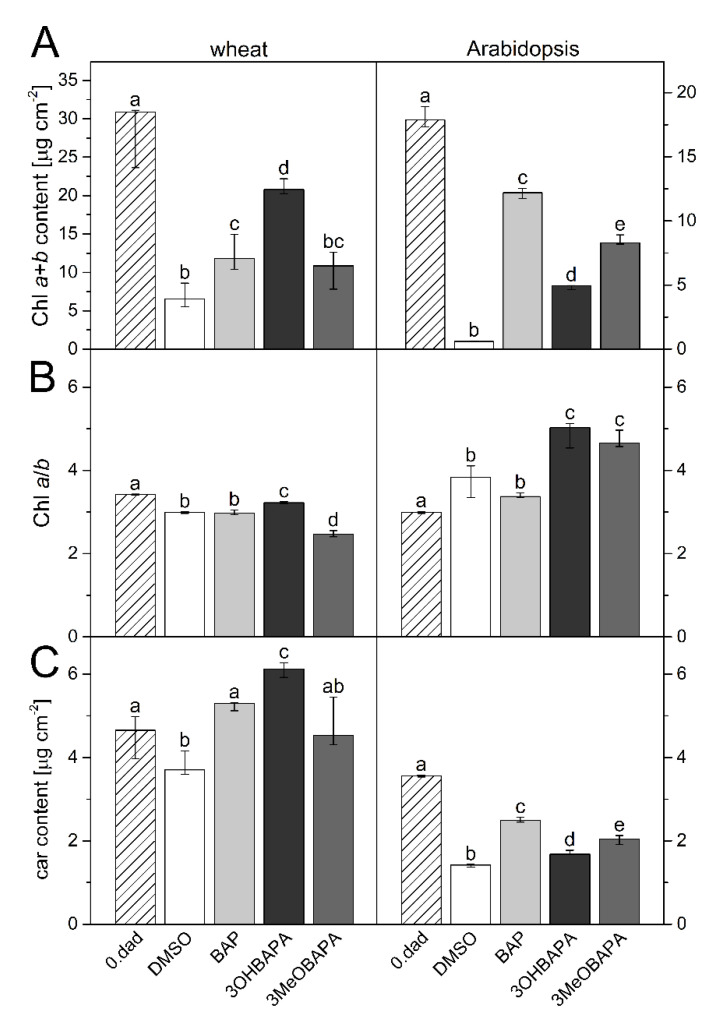
(**A**) Chlorophyll (Chl) *a*+*b* content; (**B**) Chl *a*/*b* ratio and (**C**) carotenoid (car) content in control (0. dad) and detached leaves of wheat and Arabidopsis after 6 days of dark incubation in 0.1% DMSO or 10-µmol·L^−1^ solutions of 6-benzylaminopurine (BAP), 3OHBAPA and 3MeOBAPA. Medians and quartiles are shown (*n* = 5). Different letters indicate a statistically significant difference between treatments within a plant species (*p* < 0.05; Student’s unpaired *t*-test).

The decrease in photosynthetic pigment content, deterioration of chloroplasts, and the loss of photosynthetic activity are typical phenomena of leaf senescence [1,2,3,7]. In our case, after six days of dark-induced senescence of detached wheat and Arabidopsis leaves, the content of Chl decreased significantly (Figure 2A). In line with our previous results [26], the decline in Chl content was more pronounced in the case of Arabidopsis leaves, which reflected a higher progression of senescence compared to wheat (Figure 2A). A similar trend was observed in the decrease in carotenoid content (Figure 2C). The relative rate of degradation of Chl *a* and Chl *b* differed between the leaves of wheat and Arabidopsis, as evidenced by different changes in the Chl *a*/*b* ratio (Figure 2B). The decrease in the ratio found in wheat indicated a higher degradation of Chl *a*, while in the Arabidopsis leaves Chl *b* degraded faster, which resulted in a higher Chl *a*/*b* ratio (Figure 2B). 

**Figure 3:**
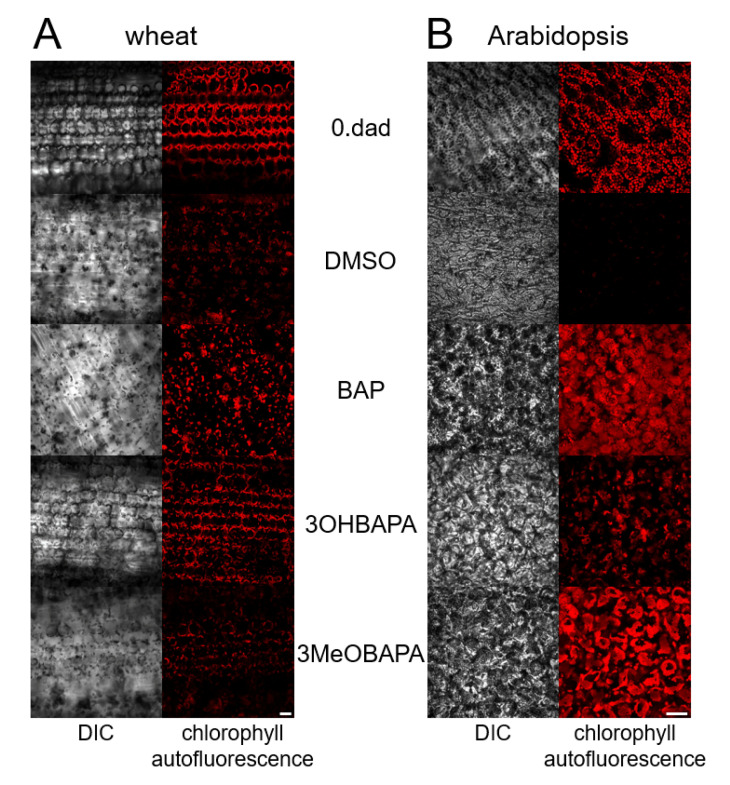
Confocal micrographs of freshly detached (0. dad) (**A**) wheat and (**B**) Arabidopsis leaves and leaves incubated in 0.1% DMSO or 10-µmol·L^−1^ solutions of BAP, 3MeOBAPA and 3OHBAPA in the dark for 6 days. Scale bars represent 50 µm. DIC, differential interference contrast.

Since the progressive decline in photosynthetic pigment contents suggests chloroplast degradation, confocal microscopy was performed to compare the chloroplast integrity in freshly detached leaves (“0. dad”) and in the leaves senescing for six days in the dark (“DMSO”; Figure 3). In senescing wheat leaves, the chloroplasts were still partially preserved, although their deterioration and aggregation was apparent (Figure 3A). In Arabidopsis, chloroplast autofluorescence was no longer detectable (Figure 3B), suggesting chloroplast degradation, which implies, together with apparent impairment of tissue structure, more advanced senescence compared to wheat. 

**Figure 4:**
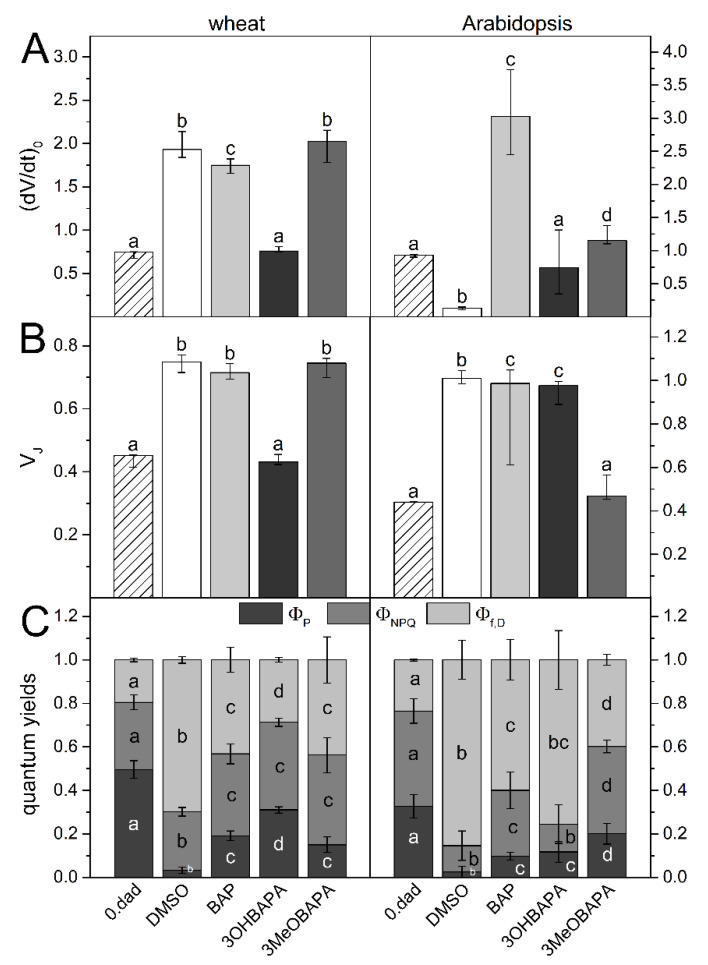
(**A**) The initial slope of the O–J Chl fluorescence rise (dV/dt)_0_, (**B**) the relative variable fluorescence at the J step (V_J_) and (**C**) quantum yields of photosystem II (PSII) photochemistry (Φ_P_), regulatory non-photochemical quenching (Φ_NPQ_) and non-regulatory dissipation processes (Φ_f,D_) in control (0. dad) and detached leaves of wheat and Arabidopsis after 6 days of dark-incubation in 0.1% DMSO or 10-µmol·L^−1^ solutions of BAP, 3OHBAPA and 3MeOBAPA. Medians and quartiles are presented (*n* = 5–12). Different letters indicate a statistically significant difference between treatments within a plant species (*p* < 0.05; Student’s unpaired *t*-test).

The different extent of senescence in wheat and Arabidopsis was also manifested at the level of PSII function, whose maintenance was estimated from the measurement of Chl fluorescence parameters (Figure 4). We measured Chl fluorescence induction transient (OJIP curve) to monitor the rate of excitation supply to reaction center (RCII), reduction of primary electron acceptor of PSII (Q_A_), and subsequent electron transport behind Q_A_ [28] during the first (milli)seconds of illumination of dark-adapted leaves. In wheat leaves, both (dV/dt)_0_ and V_J_ parameters increased (Figure 4A,B), indicating the acceleration of excitation supply to RCII and Q_A_ reduction and inhibition of electron transport behind Q_A_. Unlike in wheat, a decrease in (dV/dt)_0_ was found in Arabidopsis leaves, while V_J_ increased to its maximal value (=1). These changes indicate a strong impairment of PSII, involving minimal delivery of excitations to RCII as well as extreme inhibition of electron transfer from Q_A_^−^. In the senescent Arabidopsis leaves, the inhibition of the excitation supply to RCII could be partially related to the increased Chl *a*/*b* ratio (Figure 2B), which reflects higher degradation of light-harvesting complexes of PSII (LHCII) compared to RCII [29].

Further information on senescence-induced changes in the PSII function was obtained by measurement of quantum yields Φ_P_, Φ_NPQ_ and Φ_f,D_. These parameters, measured during exposition of leaves to actinic light, reflect the partitioning of absorbed light energy for PSII photochemistry (Φ_P_) and for regulated (Φ_NPQ_) and non-regulated (Φ_f,D_) non-photochemical processes [30]. Steady-state values of Φ_P_, Φ_NPQ_, and Φ_f,D_ measured after a 7-min exposition of the senescent leaves to actinic light are presented (Figure 4C). A higher decrease in Φ_P_ and Φ_NPQ_, and a more pronounced increase in Φ_f,D_ was found in Arabidopsis compared to wheat (Figure 4C). These results again indicate very strong impairment of PSII function in senescent Arabidopsis leaves connected with the low ability of photosynthetic apparatus to regulate dissipation of excess light energy, which subsequently can result in oxidative damage [31].

### 2.2. Comparison of the Anti-Senescence Activity of BAP and CK Arabinosides in Wheat and Arabidopsis Leaves

It is well known that the senescence-induced changes can be effectively delayed by the exogenous application of CKs. The aromatic cytokinin BAP is known to be stable in plants and highly active in delaying senescence (e.g., [3,5,8,9]). In line with the previously published results, the exogenous application of BAP slowed down the senescence-induced decrease in Chl and carotenoid content and partially suppressed deterioration of chloroplasts in both wheat and Arabidopsis (Figure 2 and Figure 3). However, the effect of BAP on parameters reflecting PSII function during the initial phase of transition of photosynthetic apparatus from a dark- to light-adapted state (the OJIP transient) was not so convincing. The only distinct effect of BAP was found for (dV/dt)_0_ in Arabidopsis. Unlike in the mock-treated leaves (“DMSO”), in the BAP-treated leaves the parameter (dV/dt)_0_ increased pronouncedly, indicating faster excitation delivery, probably due to the suppression of the preferential degradation of LHCII observed in the mock-treated Arabidopsis leaves. A minimal protective effect of BAP on excitation delivery to RCII, reduction in Q_A_ and electron transport behind Q_A_ was found in the wheat leaves (Figure 4A,B). A similar effect of BAP has already been reported by Špundová [32] in wheat, but only 7 days after leaf detachment. In leaves senescing for 4 days, BAP significantly protected electron transfer behind Q_A_, as indicated by the non-increased V_J_. Since a pronounced protective effect of BAP on excitation delivery to RCII (dV/dt)_0_ and electron transport behind Q_A_ (V_J_) was also found by Janečková [3] in detached leaves of barley senescing for 4 days, it seems that BAP protects these photosynthetic reactions especially at earlier stages of senescence and later its effect weakens. 

The senescence-induced changes in the partitioning of absorbed light energy were partially reduced in the BAP-treated leaves. The PSII photochemistry and regulatory non-photochemical quenching were more effective and the increase in the yield of non-regulated energy dissipation was lower compared to the mock-treated leaves (Figure 4C). This protective effect of BAP on PSII photochemistry corresponds with the maintenance of the maximal quantum yield of PSII photochemistry (F_V_/F_M_) found in our previous work [26]. 

Based on the estimation of overall Chl content in detached leaves of wheat and Arabidopsis, we have recently found that CK arabinosides have similar or even higher anti-senescence activity than BAP [25,26]. Two of them, 3OHBAPA and 3MeOBAPA derivatives, have been found to have a strong positive effect on F_V_/F_M_ (measured in the dark-adapted state of the senescent leaves) [26]. Interestingly, this effect differed quantitatively in wheat and Arabidopsis. 3OHBAPA had a higher protective effect in wheat, while 3MeOBAPA was more effective in Arabidopsis. In both plant species, the CK arabinosides were more effective in the maintenance of PSII function than BAP [26].

In this work, we have compared the effect of 3OHBAPA, 3MeOBAPA and BAP on senescence-induced changes in other parameters that characterize the status of photosynthetic apparatus. Among the treated senescing leaves, relative differences in carotenoid contents corresponded to those of Chl (compare Figure 2A,C). In wheat, the carotenoid content was maintained similarly in BAP- and 3MeOBAPA-treated leaves, whereas in leaves treated with 3OHBAPA many more carotenoids (even more than in the freshly detached leaves) were found, confirming the more positive effect of this CK arabinoside. In Arabidopsis, the efficiency of 3MeOBAPA was lower in comparison with BAP, but higher when compared to 3OHBAPA (Figure 2C). Similar trends were found at the level of chloroplast integrity, evaluated by confocal microscopy (Figure 3). In the case of wheat, senescence-induced deterioration of chloroplasts was similar in the BAP- and 3MeOBAPA-treated leaves, whereas in the leaves treated by 3OHBAPA chloroplasts were better preserved (Figure 3A). On the contrary, in Arabidopsis, the protective effect of 3OHBAPA was the weakest (Figure 3B).

The senescence-induced impairment of PSII function was suppressed almost completely by 3OHBAPA in wheat and 3MeOBAPA in Arabidopsis. The excitation supply to RCII, Q_A_ reduction and electron transport behind Q_A_ were highly maintained, as indicated by the unchanged values of (dV/dt)_0_ and V_J_ compared to non-senescent leaves (Figure 4A,B). Interestingly, in the 3MeOBAPA-treated leaves of Arabidopsis, the excitation supply into RCII was not accelerated (as in the BAP-treated leaves, Figure 4A), which corresponds to a higher degradation of LHCII compared to RCII, indicated also by the increased Chl *a*/*b* ratio (Figure 2B). This result is in line with the RNA-seq gene expression analysis in our previous study, where we have shown that the genes encoding LHCII components were downregulated in 3MeOBAPA-treated leaves [26]. The absence of increased delivery of excitations to RCII could contribute to the protection of photosynthetic apparatus from excess excitations and to the maintenance of the partitioning of absorbed light energy documented by minimal changes of Φ_P_, Φ_NPQ_ and Φ_f,D_ (Figure 4C). Interestingly, CK ribosides synthesized by Vylíčilová [11] caused the upregulation of LHCII genes and an increase in the relative abundance of LHCII [11], implying a different mechanism of action of CK arabinosides and CK ribosides.

In summary, 3OHBAPA had the highest anti-senescence activity in wheat, followed by 3MeOBAPA and BAP, whose activity was similar. In Arabidopsis, 3MeOBAPA was less efficient than BAP in the maintenance of photosynthetic pigment content, but it was more efficient in the maintenance of PSII function. At the same time, when compared to 3OHBAPA, 3MeOBAPA had higher anti-senescence activity, as documented by the changes in all studied parameters.

It has been shown that different activity of anti-senescence compounds can be associated with their influence on ethylene production in the detached dark-incubated leaves [27]. It is known that the exogenous application of CKs stimulates the production of ethylene [33,34]. As opposed to the positive effect of CKs on plant and leaf longevity, ethylene is known for being the accelerator of senescence [35]. Thus, the rate of the stimulation of ethylene production by exogenously applied CK can co-determine a resultant senescence-delaying effect—i.e., the anti-senescence activity of CK.

**Figure 5:**
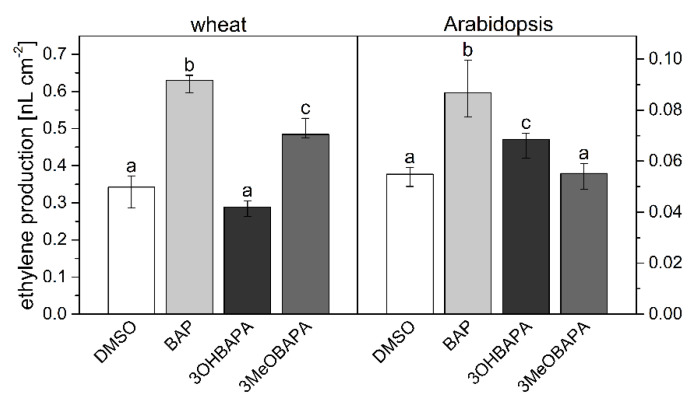
Ethylene production in detached leaves of wheat and Arabidopsis incubated in 0.1% DMSO or 10-µmol·L^−1^ solutions of BAP, 3OHBAPA and 3MeOBAPA and kept sealed in 8-mL vials in darkness for 6 days. Medians and quartiles are presented (*n* = 5–9). Different letters indicate a statistically significant difference between treatments within a plant species (*p* < 0.05; Student’s unpaired *t*-test).

We have evaluated the effect of the compounds on ethylene production in wheat and Arabidopsis leaves. As expected, the ethylene production in BAP-treated leaves was higher compared to the mock-treated leaves (Figure 5). Interestingly, no stimulation of ethylene production was observed in the wheat leaves treated by 3OHBAPA and in Arabidopsis leaves treated by 3MeOBAPA. A moderately stimulated ethylene production was observed in the 3MeOBAPA-treated wheat leaves and 3OHBAPA-treated leaves of Arabidopsis (Figure 5). Thus, the differences in the rate of ethylene production corresponded generally to the quantitative differences of anti-senescence activity between BAP and BAPAs, as well as between 3OHBAPA and 3MeOBAPA: the lower the ethylene production, the higher the anti-senescence activity.

### 2.3. Species-Specific Effects of BAPAs Treatment in the Protection Against Induced Oxidative Damage

It has been shown that the inhibition of ethylene production not only suppresses leaf senescence, but it also enhances the tolerance of plants to abiotic stresses, including H_2_O_2_- and high light (HL)-induced oxidative stress [36]. As no stimulation of ethylene production was found in wheat leaves treated by 3OHBAPA and Arabidopsis leaves treated by 3MeOBAPA (Figure 5), we hypothesized that the mentioned BAPA treatments could provide a more efficient protection of leaves against oxidative damage induced by H_2_O_2_- and HL-treatment than the BAP treatment.

**Figure 6:**
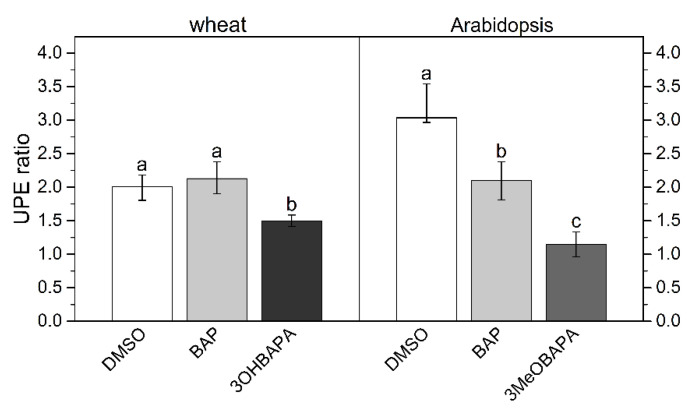
Ratio of ultra-weak photon emission (UPE) intensity after/before application of H_2_O_2_ in detached leaves of wheat and Arabidopsis dark-incubated for 6 days in 0.1% DMSO or 10-µmol·L^−1^ solutions of BAP, 3OHBAPA or 3MeOBAPA. Medians and quartiles are presented (*n* = 4). Different letters indicate a statistically significant difference between treatments within a plant species (*p* < 0.05; Student’s unpaired *t*-test).

For a comparison of the protective effect of CK arabinosides and BAP, senescent leaves were exposed to exogenous H_2_O_2_ (5 mmol·L^−1^) and the level of oxidative damage was evaluated using ultra-weak photon emission (UPE) [37]. In the mock-treated leaves, the H_2_O_2_ treatment caused pronounced oxidative damage as indicated by the high UPE ratio (Figure 6). In wheat, the protective effect indicated by the lower UPE ratio was observed only in leaves treated with 3OHBAPA. In Arabidopsis, both BAP and 3MeOBAPA reduced the oxidative damage, 3MeOBAPA being more effective (Figure 6).

**Figure 7:**
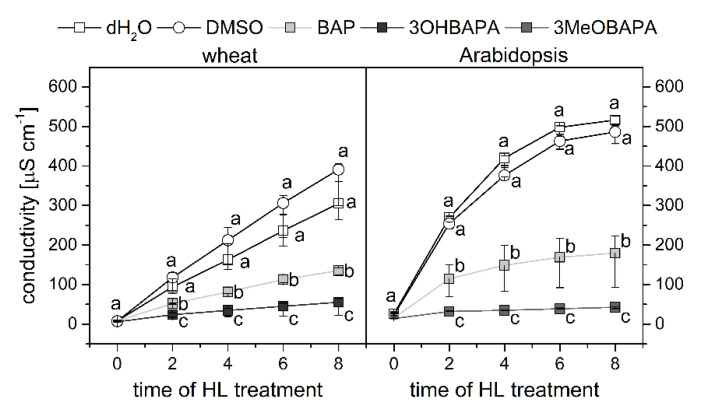
Conductivity as a measure of cell membrane damage in wheat- and Arabidopsis-detached leaves incubated in deionized water (dH_2_O), 0.1% DMSO, 10-µmol·L^−1^ solutions of BAP, 3OHBAPA or 3MeOBAPA. Before high-light treatment (HL; 500 µmol photons·m^−2^·s^−1^) for up to 8 h leaves were incubated in the dark for 6 days. Medians and quartiles are presented (*n* = 7). Different letters indicate a statistically significant difference between treatments within a plant species and particular time point (*p* < 0.05; Student’s unpaired *t*-test).

**Figure 8:**
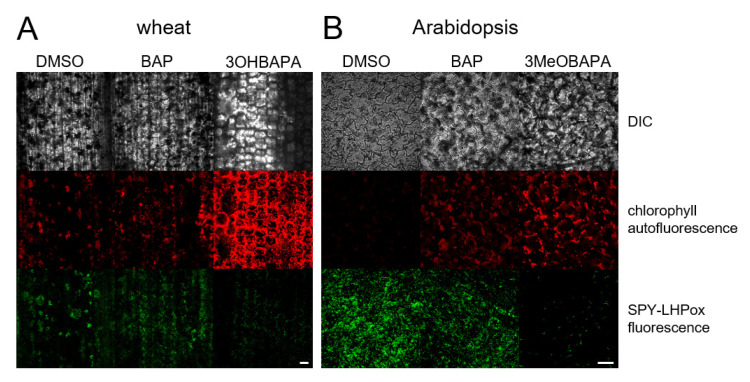
In vivo imaging of lipid hydroperoxides (LOOHs) using fluorescent probe SPY-LHP in (**A**) wheat and (**B**) Arabidopsis leaves kept in the dark for 6 days and subsequently exposed to high light (500 µmol photons·m^−2^·s^−1^) for 4 h. Detached leaves were incubated in 0.1% DMSO or 10-µmol·L^−1^ solutions of BAP, 3OHBAPA or 3MeOBAPA. Scale bars represent 50 µm. DIC, differential interference contrast.

**Figure 9:**
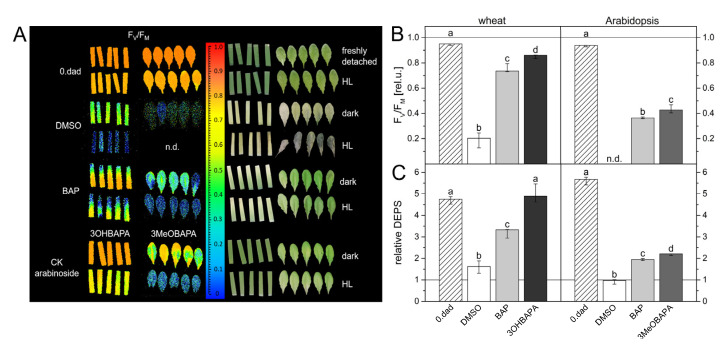
(**A**) Imaging of maximum quantum yield of PSII photochemistry (F_V_/F_M_) before and after high-light treatment (HL; 500 µmol photons·m^−2^·s^−1^ for 4 h) of freshly detached leaves and leaves incubated for 6 days in 0.1% DMSO or 10-µmol·L^−1^ solutions of BAP, 3OHBAPA or 3MeOBAPA; (**B**) the relative F_V_/F_M_ values and (**C**) relative de-epoxidation state of xanthophylls (DEPS) in detached wheat and Arabidopsis leaves. The relative F_V_/F_M_ and DEPS were calculated as a ratio of values measured after and before exposure to high light. In the column graphs, medians and quartiles estimated from presented values of individual leaves are shown (*n* = 5). Different letters indicate a statistically significant difference between treatments within a plant species (*p* < 0.05; Student’s unpaired *t*-test).

In the following experiment, we exposed the senescent leaves to HL (500 µmol photons·m^−2^·s^−1^) and estimated cell membrane damage using the measurement of ion leakage by conductivity, accumulation of lipid hydroperoxides (LOOHs; as primary products of lipid peroxidation and markers of oxidative damage) by confocal microscopy, and maintenance of the maximal quantum yield of PSII photochemistry (F_V_/F_M_), which reflects the extent of photoinhibition or photodamage of PSII. The exposure of senescing leaves to HL led to a strong oxidative damage, as indicated by progressive deterioration of cell membranes during 8 h of HL treatment (Figure 7), and by LOOH accumulation (Figure 8) and photoinhibition of PSII (Figure 9) after 4 h of HL treatment. The oxidative damage was suppressed by the exogenous application of BAP, although its protective effect was quite low. The HL-induced membrane deterioration and PSII photoinhibition were attenuated in the BAP-treated leaves of both wheat and Arabidopsis; LOOH accumulation was reduced only in Arabidopsis (Figure 7, Figure 8 and Figure 9).

**Figure 10:**
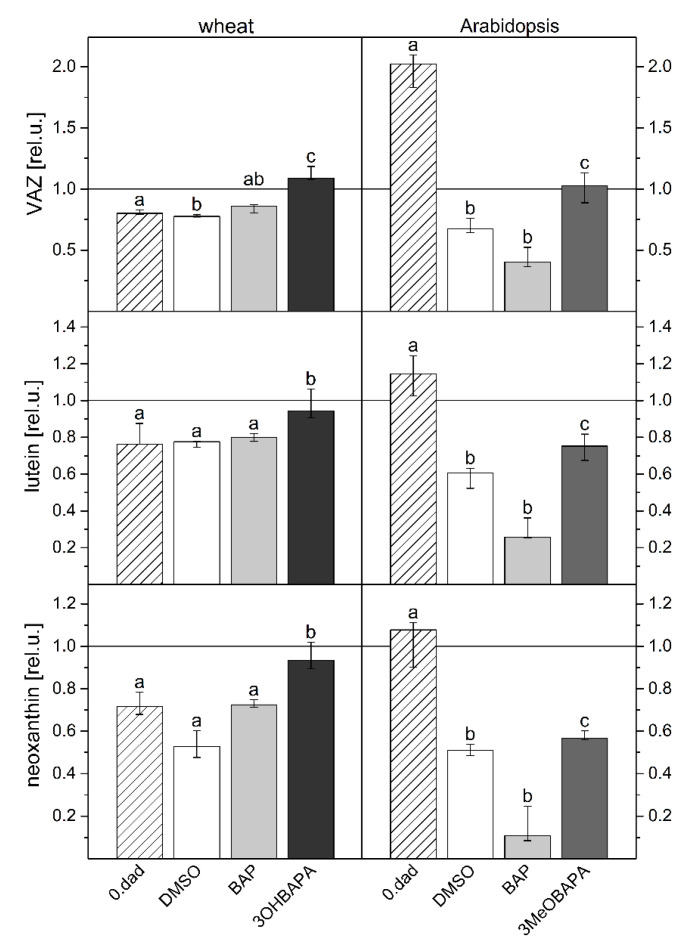
Violaxanthin, antheraxanthin, zeaxanthin (VAZ), lutein, and neoxanthin content (relatived to the value before high-light treatment) in control (0. dad) and detached leaves of wheat and Arabidopsis incubated in 0.1% DMSO or 10-µmol·L^−1^ solutions of BAP, 3OHBAPA or 3MeOBAPA. Leaves were kept in the dark for 6 days and subsequently exposed to high light (500 µmol photons·m^−2^·s^−1^ for 4 h). Medians and quartiles are presented (*n* = 4–5). Different letters indicate a statistically significant difference between treatments within a plant species (*p* < 0.05; Student’s unpaired *t*-test).

As expected, 3OHBAPA in wheat and 3MeOBAPA in Arabidopsis were more effective in the protection of senescing leaves from oxidative damage than BAP. BAPAs markedly reduced the HL-induced cell membrane damage (Figure 7), accumulation of LOOHs (Figure 8), as well as PSII photoinhibition (Figure 9). We suppose that in the case of HL-treated wheat leaves, the high protective effect of 3OHBAPA can be mainly ascribed to the completely maintained xanthophyll cycle (Figure 9C) together with the high content of xanthophylls (Figure 10), both of which are known to be important for photoprotection [38,39].

In Arabidopsis, the xanthophyll cycle was only moderately stimulated in the 3MeOBAPA-treated leaves exposed to HL (Figure 9), so the protective effect of 3MeOBAPA seems to be associated with the lower excitation delivery to RCII (due to the decreased LHCII/RCII ratio) (Figure 4A) and to the maintenance of xanthophylls during HL treatment (Figure 10). On the other hand, the lower protection of the BAP-treated senescent leaves could be related to the increased excitation supply to RCII (Figure 4A) due to the maintained LHCII/RCII ratio. Under HL, photosynthetic apparatus in the BAP-treated senescent leaves was most probably overexcited, which could contribute to the lower protective effect of BAP against oxidative damage. This finding is consistent with our previous work, where we have shown that under excessive light conditions, the protective action of exogenous CKs on senescent leaves can switch to damaging [7,21] due to the overexcitation of photosynthetic apparatus and oxidative damage [7]. 

The effective anti-oxidative protection by BAPAs could be related to the upregulation of *JUB1, ELIP1* and *ELIP2* detected in the 3MeOBAPA-treated Arabidopsis leaves (Table S1). The ROS-responsive transcription factor *JUB1* is considered to be a central longevity regulator in Arabidopsis that acts through the modulation of H_2_O_2_ level in cells [40]. It has been found that the overexpression of *JUB1* not only delays senescence, but also enhances tolerance to abiotic stresses [40]. 

Early-light-induced proteins (ELIPs) belong to the family of pigment-protein LHCs and fulfill a protective function and prevent oxidative damage under conditions of excess light [41]. The photoprotective function of ELIPs is based on the transient binding of free Chl and was originally thought to be important predominantly for developing leaves (e.g., [42]). However, the protective role of ELIPs was also reported during senescence, although their upregulation was conditioned by the presence of light [43] and/or correlated with light intensity [44]. Surprisingly, 3MeOBAPA upregulated *ELIPs* even in the dark, indicating that some aspects of 3MeOBAPA actions are similar to the effect of increased light. Additionally, the reduction in the LHCII/RCII ratio, observed in 3MeOBAPA-treated leaves, is typical for leaf acclimation to high-light intensities. This 3MeOBAPA-induced response may help leaves to cope with subsequent (photo)oxidative stress. Additionally, 3MeOBAPA has been shown to protect human dermal fibroblasts from UV-A and UV-B treatment [25], thus the protective activity of this compound may be more universal. The mechanism of this 3MeOBAPA effect remains to be elucidated.

### 2.4. On the Mechanism of Specific Anti-Senescence Activity of CK Arabinosides 

In our previous work, we have shown that the unique mode of CK arabinosides’ action differs from that of CKs. The CK arabinosides were shown to have only low activity in the *Amaranthus* and callus CK bioassays [25,26]. However, their activity in the senescence bioassay was high, despite the fact that in vitro they did not activate the AHK3 receptor [26], which is considered to be the main receptor involved in CK-mediated delay of senescence [2,45] and protection during HL stress [46]. The RNA-seq gene expression analysis performed by Bryksová [26] in Arabidopsis 3MeOBAPA-treated leaves revealed a broad range of transcriptomic changes typical for the activation of the PAMP-response: downregulation of a number of photosynthetic genes and upregulation of many defense genes. A similar response can be expected in BAPAs-treated wheat and barley plants, as their enhanced resistance to pathogens was observed in field trial experiments [26].

The upregulation of genes of defense regulons in the 3MeOBAPA-treated Arabidopsis leaves included jasmonate/ethylene-driven upregulation of plant defensins and was accompanied by a significant elevation of endogenous levels of jasmonic acid (JA) and its metabolites [26]. However, as we have demonstrated here, the ethylene production in the BAPA-treated leaves was low, which might be one of the main reasons for the BAPAs’ high anti-senescence activity, as ethylene is known to promote leaf senescence [35]. It has been reported that the key transcription factor of the ethylene signaling pathway *EIN3* and its downstream target *ORE1* directly activate genes of Chl degradation. *ORE1* also directly promotes ethylene synthesis and ethylene in turn accelerates leaf senescence through *EIN3*–*ORE1* [47]. *EIN3* and *ORE1* were downregulated by 3MeOBAPA (Table S1), which indicates that the attenuation of this loop is involved in the anti-senescence activity of 3MeOBAPA. This activity is also evidenced by the downregulation of other positive regulators of senescence, such as *SAG12*, *ORE3*, and *ORE9* (Table S1). In summary, in this work we show for the first time that the anti-senescence effect of CK arabinosides in Arabidopsis may be related to specific downregulation of the *EIN3*–*ORE1* pathway which has been previously shown to be involved in leaf degreening through Chl catabolic gene regulation [47]. 

Similarly to ethylene, JA is considered to be a positive regulator of leaf senescence. As mentioned, the level of JA and its metabolites was found to be increased in the 3MeOBAPA-treated Arabidopsis leaves [26]. However, this increase was only temporary—it was observed after 48 h of the treatment [26], while after 4 days the JA level was lower than that in the mock-treated leaves (unpublished data). The transient increase in endogenous JA content appears to be involved in triggering the defense response by 3MeOBAPA and does not stimulate, but rather suppresses, leaf senescence. This hypothesis is in line with the upregulation of the JA-signalling repressors *JAZ7* and *JAZ 8* (Table S1), as JAZ7 is known to be a specific negative regulator of dark-induced leaf senescence and together with JAZ8 suppresses the activation of *SAGs* including *SAG12* [48,49].

The specific effects of CK arabinosides described in this work represent further evidence that they act through a mechanism different from that of classical CKs. Because they have high anti-senescence activity and protective effects under stress conditions and simultaneously do not impair root development, they are promising substances for plant protection. However, further study is needed to elucidate the exact mechanism of their action. 

## 3. Materials and Methods 

### 3.1. Plant Material 

Plants of spring wheat (*Triticum aestivum* L. cv. Aranka) were grown on an artificial medium composed of perlite and Hoagland’s solution in a growth chamber at 25 °C under a 16-h light (120 µmol photons·m^−2^·s^−1^)/8-h dark cycle for 7 days. Then segments were cut off from the primary leaves, 4 cm from the leaf tip. The basal end of the leaf segment was placed into a well of 96-well plate with 200 µL of 10-µmol·L^−1^ solutions of BAP, 3OHBAPA or 3MeOBAPA dissolved in 0.1% dimethylsulfoxide (DMSO; Sigma-Aldrich, St. Louis, MO, USA) or with 0.1% DMSO solution in deionized water (mock control). 3OHBAPA and 3MeOBAPA (Figure 1) were prepared as described previously [25,26]. The segments were kept in darkness at 24 °C for 6 days. Plants of *Arabidopsis thaliana* L. (Col-0) were grown in a soil (Potgrond H, Klasmann-Deilmann, Geeste, Germany) in a growth chamber under an 8-h light (120 µmol photons·m^−2^·s^−1^)/16-h dark cycle and at 22 °C/20 °C for 6 weeks. Then the 7th and 8th rosette leaves were detached from the plants and incubated in a six-well plate with 5 mL of the solutions described above. Freshly detached Arabidopsis and wheat leaves were used as control (0. day after detachment; dad).

### 3.2. High-Light Treatment 

After six days in the dark, wheat leaf segments and Arabidopsis leaves incubated in the solutions described above were exposed to high-light (HL; white light, 500 µmol photons·m^−2^·s^−1^) for up to 8 h to induce photo-oxidative stress. In the case of wheat, edges of the segments were cut off and the rectangular middle part (2 cm long) of the leaf segment was placed into solutions prior to the HL treatment. 

### 3.3. Determination of Chlorophyll, Carotenoid and Xanthophyll Content

Following estimation of their area, leaf samples were frozen in liquid nitrogen, stored at −80 °C and subsequently homogenized using 80% acetone and a small amount of MgCO_3_. Homogenate was centrifuged at 3600× *g* for 15 min at 4 °C. The supernatant was used for spectrophotometric (Unicam UV 500, Thermo Spectronics, Cambridge, UK) estimation of Chl and carotenoid contents according to Lichtenthaler [50] and for quantification of xanthophylls (violaxantin, V; anteraxantin, A; zeaxantin, Z; neoxantin; lutein) by a reversed-phase high-performance liquid chromatography (HPLC) using Alliance e 2695 HPLC System (Waters, Milford, MA, USA) equipped with 2998 Photodiode Array detectors. The separation was carried out using a gradient system (1.5 mL·min^−1^ at 25 °C) on a LiChrospher^®^ 100 RP-18 (5 µm) LiChroCART^®^ 250-4 (Merck, Darmstadt, Germany). Quantification was performed by absorbance at the following wavelength: 437 (neoxanthin), 441 (violaxanthin), 446 (antheraxanthin), 447 (lutein), and 454 nm (zeaxanthin) using standards purchased from DHI Lab Products (Hørsholm, Denmark). The de-epoxidation state of xanthophylls (DEPS) was calculated as DEPS = (A + Z)/(V + A + Z) [51].

### 3.4. Confocal Laser Scanning Microscopy

Confocal microscopy was performed on detached leaves kept in the dark for 6 days and on leaves subsequently exposed to HL for 4 h. Using fluorescent probe SPY-LHP (swallow-tailed perylene derivative; Dojindo Molecular Technologies, Rockville, MD, USA), lipid hydroperoxides (LOOHs) were localized. Wheat and Arabidopsis leaves were cut into pieces (approximately 2 mm × 2 mm) and incubated in 50 µmol·L^−1^ SPY-LHP in HEPES buffer (10 m µmol·L^−1^, pH 7.5) in the dark at room temperature for 30 min. Afterward, the samples were visualized by a confocal microscope (FV1000, Olympus Czech Group, Prague, Czech Republic). The excitation of fluorochrome was performed using a 488-nm line of argon laser. The signal was detected through a 505–550-nm emission filter. The morphology of tissues was visualized by a 405-nm diode laser excitation in transmitted light detection module with differential interference contrast (DIC) filters, while chloroplasts by a 543-nm helium–neon laser excitation with emission recorded with a 655–755-nm bandpass filter. The proper intensity of the laser was set according to unstained samples at the beginning of each experiment [52]. During the image postprocessing in FV10-ASW 4.0 Viewer software (Olympus Czech Group, Prague, Czech Republic), the contrast of the SPY-LHP channel was increased to 3 in the case of wheat and to 4 in the case of Arabidopsis. Five replicates were performed and representative images are presented.

### 3.5. Chlorophyll Fluorescence Parameters (PSII Functioning)

Chl fluorescence induction transient (OJIP curve) and Chl fluorescence quenching analysis were measured at room temperature from the adaxial side of the leaves using a Plant Efficiency Analyser (Hansatech Instruments, Norfolk, UK) and FluorCam imaging system (PSI, Drásov, Czech Republic), respectively. The control leaves and HL-treated leaves were dark-adapted for 30 min prior to the measurement. In the case of senescent leaves, dark adaptation was not necessary due to their incubation in the dark. 

The OJIP transient was measured in the middle of leaf segments with excitation light (red light, 4300 µmol photons·m^−2^·s^−1^) lasting 1 s. The initial slope of the O–J Chl fluorescence rise (dV/dt)_0_ and the relative variable fluorescence at the J step (V_J_) were evaluated. The (dV/dt)_0_ parameter, defined as the maximal rate of accumulation of closed (i.e., reduced) RCII, was calculated as (dV/dt)_0_ = 4 (F_300μs_ – F_50μs_)/F_V_, where F_300μs_ and F_50μs_ are fluorescence intensities at the indicated times of the transient, and F_V_ is variable fluorescence (F_V_ = F_P_ – F_0_, where F_P_ is fluorescence intensity at P step and F_0_ is minimal fluorescence) [28]. The V_J_ parameter reflects a fraction of reduced RCII and was calculated as V_J_ = (F_J_ – F_0_)/F_V_, where F_J_ is fluorescence intensity at 2 ms (J-step). 

A FluorCam imaging system was used for the measurement of quantum yields Φ_P_, Φ_NPQ_ and Φ_f,D_ reflecting the proportion of three different types of energy usage by PSII (their sum equals 1; [30]) in light-adapted state of senescent leaves, and for measurement of changes in maximum quantum yield of PSII photochemistry (F_V_/F_M_ = (F_M_ − F_0_)/F_M_) after HL treatment (4 h). The effective quantum yield of PSII photochemistry for the light-adapted state was calculated as Φ_P_ = (F_M_′ − F_t_)/F_M_′. The quantum yield of regulatory light-induced non-photochemical quenching was calculated as Φ_NPQ_ = (F_t_/F_M_′) − (F_t_/F_M_). The quantum yield of constitutive non-regulatory dissipation processes was calculated as Φ_f,D_ = F_t_/F_M_. The minimal fluorescence of the dark-adapted leaf sample (F_0_) was determined by applying several µ-seconds-long measuring flashes (red light, 0.1 µmol photons·m^−2^·s^−1^) at the beginning of the procedure. The maximal fluorescence of the dark-adapted sample (F_M_) was measured using the 1.6-s saturating pulse (white light, 850 µmol photons·m^−2^·s^−1^). After 2 min of dark relaxation the sample was exposed to actinic light (red light, 200 µmol photons·m^−2^·s^−1^) and series of saturating pulses (the 1st pulse at the 3rd second of actinic light, 6 pulses in a 23-s interval, 3 pulses in a 47-s interval and the last 2 pulses in a 70-s interval) to measure the maximal fluorescence in the light-adapted sample (F_M_′). The actual fluorescence signal at the time *t* of actinic illumination (F_t_) was measured immediately prior to the application of saturating pulse. The last measured (steady-state) values of Φ_P_, Φ_NPQ_ and Φ_f,D_ are presented. 

### 3.6. Ethylene Production

The detached leaves of wheat and Arabidopsis were put into 10-µmol·L^−1^ solutions of BAP, 3OHBAPA, 3MeOBAPA, or into 0.1% DMSO solution in distilled water and kept sealed in 8-mL vials in darkness for 6 days. Subsequently, 1.5 mL of air was collected from each vial and reduced to 1 mL before injection to gas-chromatograph Agilent GC 6890 (Agilent Technologies, Santa Clara, CA, USA) equipped with a flame ionization detector and 50-m capillary column (HP-AL/S stationary phase, 15 µm, i.d. = 0.535). The injection temperature was set to 200 °C, oven temperature to 40 °C, and the detector temperature to 220 °C. The measurements were performed in triplicate from three different test tubes of each variant. 

### 3.7. Ultra-Weak Photon Emission 

Two-dimensional imaging of ultra-weak photon emission (UPE) [53] was measured from the adaxial side of the leaves kept in the dark for 6 days before and after their treatment with 5-mmol·L^−1^ H_2_O_2_. After the first UPE measurement, the leaf segments were let to dry off for 30 min under common laboratory conditions and then put into the H_2_O_2_ solution. After 3 h of H_2_O_2_ incubation, the second UPE measurement was performed while the leaves remained in the H_2_O_2_ solution. A ratio of UPE of the leaf after and before the H_2_O_2_ incubation was estimated. The UPE was detected by a highly sensitive CCD camera VersArray 1300B (Princeton Instruments Trenton, NJ, USA) equipped with an objective (F mount Nikkor 50 mm, f/1.2, Nikon, Tokyo, Japan). The CCD element was cooled down to −104 °C to reduce the background noise, the accumulation time of each measurement was 30 min. The UPE of the leaf represents the average number of counts from the leaf surface.

### 3.8. Ion Leakage 

For the determination of the extent of ion leakage from leaf tissue as a measure of cell membrane damage, circular (diameter of 1 cm) or rectangular (2 cm long) segments were cut out from Arabidopsis leaves or wheat leaf segments, respectively. Groups of six leaf discs or eight rectangular segments (one group represents one sample) were put into 6-well plates containing 3.5 mL of deionized water and incubated under HL (500 µmol photons·m^−2^·s^−1^) for up to 8 h to induce oxidative damage in cell membranes. The conductivity of the solutions was measured after 2, 4, 6 and 8 h of the HL treatment with a conductivity meter (GMH 3430, Greisinger, Regenstauf, Germany). As DMSO is known to affect the membrane fluidity and permeability for ions, deionized water was used as a mock control as well to exclude effect of DMSO on the membrane permeability.

## Supplementary Materials

Supplementary materials can be found at https://www.mdpi.com/1422-0067/21/21/8109/s1.

## Abbreviations

A	antheraxanthin
AHK	Arabidopsis histidine kinase
BAP	6-benzylaminopurine
car	carotenoid
Chl	chlorophyll
CK	cytokinin
dad	day after detachment
DEPS	de-epoxidation state of xanthophylls
DIC	differential interference contrast
DMSO	dimethylsulfoxide
(dV/dt)_0_	the initial slope of the O–J Chl fluorescence rise
F_V_/F_M_	the maximum quantum yield of photosystem II photochemistry
HL	high light
JA	jasmonic acid
LHCII	light-harvesting complexes of photosystem II
LOOH	lipid hydroperoxide
OJIP	chlorophyll fluorescence induction transient
PSII	photosystem II
Q_A_	primary electron acceptor of photosystem II
RCII	reaction center of photosystem II
*SAG*	senescence-associated gene
UPE	ultra-weak photon emission
V	violaxanthin
V_J_	the relative variable fluorescence at the J step of OJIP curve
Z	zeaxanthin
3OHBAPA	6-(3-hydroxybenzylamino)-9-β-d-arabinofuranosylpurine
3MeOBAPA	6-(3-methoxybenzylamino)-9-β-d-arabinofuranosylpurine
Φ_f,D_	the quantum yield of constitutive non-regulatory dissipation processes
Φ_NPQ_	the quantum yield of regulatory light-induced non-photochemical quenching
Φ_P_	the effective quantum yield of photosystem II photochemistry (for the light-adapted state)
