# Supplementary material for: The Anti-Senescence Activity of Cytokinin Arabinosides in Wheat and Arabidopsis Is Negatively Correlated with Ethylene Production
Source: Int J Mol Sci. 2020 Oct 30;21(21):8109. doi: 10.3390/ijms21218109 (PMC7662598; doi:10.3390/ijms21218109)
Supplement: Supplementary file 1 [file ijms-21-08109-s001.pdf]

Table S1 The expression level of selected genes involved in senescence regulation and protection against oxidative damage in *Arabidopsis thaliana* L. (AT, Col-0) after 48 h treatment with 10 μmol·L^{-1} 3MeOBAPA. Gene ID, name and description and log2 fold change of mock-treated and 3MeOBAPA-treated transcript abundance are listed. RNA-seq gene expression analysis was performed within our previous study [26].

ID	Log2 fold change	Gene name	description
AT5G45890	-12.35	*SAG12*	SENESCENCE-ASSOCIATED GENE 12
AT5G03280	-0.93	*EIN2, ORE3*	ETHYLENE INSENSITIVE 2, ORESARA 3
AT3G20770	-0.69	*EIN3*	ETHYLENE INSENSITIVE 3
AT5G39610	-1.30	*ORE1*	ORESARA 1
AT2G42620	-1.66	*ORE9*	ORESARA 9
AT2G43000	4.14	*JUB1*	JUNGBRUNNEN 1
AT3G22840	5.02	*ELIP1*	EARLY-LIGHT INDUCED PROTEIN 1
AT4G14690	3.71	*ELIP2*	EARLY-LIGHT INDUCED PROTEIN 2
AT1G30135	5.19	*JAZ8*	JASMONATE-ZIM-DOMAIN PROTEIN 7
AT2G34600	1.13	*JAZ7*	JASMONATE-ZIM-DOMAIN PROTEIN 8
